# A Physics-Guided Quantitative GPR Framework for Detecting Hanging Sleepers in Ballasted Railway Tracks

**DOI:** 10.3390/s26061905

**Published:** 2026-03-18

**Authors:** Wen Yang, Jie Gao, Zhi Xu

**Affiliations:** 1 China Energy Shuohuang Railway Development Co., Ltd., Cangzhou 062350, China; 11091802@ceic.com (W.Y.); 20007983@ceic.com (J.G.); 2 Hangzhou Institute of Technology, Xidian University, Hangzhou 311231, China

**Keywords:** sleeper voids, ground-penetrating radar, time-domain analysis, continuous wavelet transform, ballasted tracks, non-destructive testing

## Abstract

Sleeper voids, or hanging sleepers, in ballasted railway tracks threaten structural safety and serviceability. This study proposes a physics-guided quantitative ground-penetrating radar (GPR) framework for detecting hanging sleepers using high-frequency antennas (f≥1.5 GHz). The framework integrates signal post-processing, sleeper-region localization, time-domain peak searching with polarity consideration, and continuous wavelet transform (CWT) as auxiliary verification. By exploiting the physical geometric relationship between the sleeper and ballast interfaces, the method quantitatively estimates their elevation difference and identifies hanging sleepers according to engineering criteria. Spatial continuity constraints are further introduced to reduce false detections. Validation through gprMax simulations and field experiments demonstrates effective detection and severity assessments, providing a physically interpretable solution for automated railway inspection.

## 1. Introduction

### 1.1. Research Background and Motivation

Sleeper voids, commonly referred to as hanging sleepers, are a prevalent defect in ballasted railway tracks, characterized by gaps between the sleeper base and the ballast layer. These voids primarily result from ballast settlement, fouling, repeated train loading, and inadequate drainage [1,2,3,4]. They compromise the uniform transfer of loads from rails to the subgrade, resulting in uneven track settlement, amplified wheel–rail impact forces, and the accelerated deterioration of track components [5]. In extreme cases, sleeper voids can jeopardize operational safety, contributing to derailments or service interruptions, as evidenced by incidents in high-speed and heavy-haul railways [6]. The timely and accurate detection of sleeper voids is, therefore, critical for preventive maintenance, enhancing track reliability, minimizing downtime, and reducing maintenance costs [7]. With the continued expansion of railway networks worldwide and increasing operating speeds, there is an urgent need for efficient, non-destructive testing (NDT) techniques to support sustainable railway infrastructure management.

### 1.2. Literature Review

Railway track structures are continuously subjected to repeated dynamic loads during long-term service. These cyclic loads gradually degrade the mechanical properties of track components, including the ballast layer and subgrade. Comprehensive reviews have summarized experimental facilities and predictive models for evaluating the long-term service performance of railway subgrades under repeated traffic loads, highlighting the progressive deterioration of the ballast–subgrade system as an important mechanism of sleeper void development [8].

The existing methods for detecting sleeper voids can be broadly categorized into traditional, dynamic, and NDT approaches. Traditional methods, such as visual inspection and hammering tests, identify voids through auditory feedback (e.g., hollow sounds) or track geometry measurements [4,9,10]. These approaches have demonstrated reasonable effectiveness in detecting severe voids, with studies showing detection accuracies of up to 80% under controlled conditions [11]. However, these methods are highly subjective, labor-intensive, and limited in quantifying the void depth or extent [12].

Dynamic methods, including axle–box acceleration analysis and curvature-based techniques, facilitate real-time monitoring under in-service loads, achieving detection rates of 85–95% for voids exceeding 5 mm in depth [13,14]. These methods infer void formation based on track stiffness changes but are sensitive to ballast fouling and moisture content, which can reduce their robustness in early-stage void detection. Recent innovations, such as model-based void geometry identification from track-side rail deflections, enable the precise quantification of void geometry but require extensive calibration to account for variable track conditions [15]. Studies on unsupported sleepers under cyclic loading further reveal accelerated settlement accumulation in void zones, with elasto-plastic models indicating up to 50% higher degradation rates [16]. Despite these advancements, dynamic approaches remain vulnerable to interferences from ballast fouling, moisture, and train speed variations, often leading to false positives and challenges in early-stage detection [17]. Overall, existing methods frequently fail to provide continuous, non-intrusive assessments, underscoring the need for more advanced detection solutions to mitigate safety risks in high-speed railway systems.

NDT methods, including ultrasonic testing, impact-echo, and laser scanning, provide enhanced detection coverage without disrupting railway operations [18,19]. Impact-echo methods detect voids by analyzing stress wave propagation [20]. While these methods are more objective and less labor-intensive than traditional techniques, they suffer from limited penetration depth in fouled ballast, high equipment costs, and the need for specialized data interpretation.

Ground-penetrating radar(GPR) has been widely applied in sleeper and ballast inspection [21], leveraging electromagnetic wave reflections to detect voids [22,23]. High-frequency GPR (1–2 GHz) provides a superior resolution for shallow defects such as sleeper voids, with studies demonstrating void detection accuracies exceeding 90% based on amplitude and travel time analysis [3]. Beyond direct void identification, GPR has also been widely used for ballast condition assessment, fouling evaluation, moisture characterization, and subsurface interface inspection in railway track structures [21,23]. These studies demonstrate that GPR is one of the most promising non-destructive tools for ballast–track diagnosis because it enables rapid, continuous, and vehicle-mounted inspection without interrupting railway operation.

Time-frequency domain features, such as continuous wavelet transforms (CWT) and spectral energy distribution, have proven particularly effective in evaluating ballast defects by capturing signal dispersion and attenuation characteristics [24,25,26]. For example, research utilizing CWT on GPR signals has quantified ballast fouling levels and void extents by identifying frequency shifts and energy peaks, thereby reducing interference from moisture and background clutter [27]. Numerical simulations using tools such as gprMax have further validated these spectral responses in void scenarios [23]. From an algorithmic perspective, existing GPR-based methods for sleeper void or similar shallow-defect detection can generally be grouped into four categories:

1. Amplitude-based interpretation, in which voids are inferred from an anomalous reflection magnitude [28].
2. Travel-time analysis, where defect geometry is estimated from arrival-time differences of interface reflections [29];
3. Time–frequency feature extraction, such as CWT and spectral energy analysis, which characterizes dispersed or attenuated responses in the time–frequency domain.
4. Numerical simulation or data-driven approaches, in which electromagnetic modeling tools are used to validate defect responses and improve automated interpretation [30].

Despite these advances, the literature specifically addressing sleeper void detection remains relatively limited compared with broader ballast-condition assessment. Most existing GPR studies rely primarily on either amplitude variation or spectral-energy characteristics, whereas the direct physical relationship between sleeper support loss and the travel-time difference between reflections from the sleeper top and ballast surface has not been sufficiently exploited. In addition, integrated verification between time-domain peak localization and frequency-domain interpretation remains scarce, which reduces quantitative reliability under varying ballast conditions.

This study addresses the limitations of existing GPR methods in the precise detection of sleeper voids in ballasted tracks, particularly the lack of direct exploitation of time-domain waveform differences between sleeper tops and ballast surfaces. This study proposes a novel approach utilizing high-frequency GPR (≥1.5 GHz) spectral features, which combines time-domain peak searching with CWT for the integrated verification of time delays (Δt) and void heights (*H*). Through the establishment of adaptive thresholds based on sleeper thickness and dielectric corrections, the method enables quantitative and automated void detection with enhanced robustness against environmental interference. Validated through gprMax-based numerical simulations and field experiments, the proposed method fills a gap in current NDT practices, providing real-time, vehicle-mounted capabilities for railway maintenance.

### 1.3. Contributions

Current sleeper void detection techniques remain constrained by subjectivity, indirect stiffness inference, or amplitude-dependent interpretation. In particular, most existing GPR-based approaches primarily rely on reflection amplitude variation or spectral energy distribution, which are highly sensitive to ballast moisture, fouling conditions, and signal attenuation. These methods emphasize signal intensity or pattern characteristics, rather than directly quantifying the geometric void beneath sleepers.

It should be noted that the individual signal-processing components adopted in this study, such as time-domain peak searching, CWT, and thresholding, are not entirely new in the general field of GPR data analysis. The contribution of this work lies in integrating these techniques into a sleeper-void-oriented detection framework with explicit physical interpretation and automated quantitative decision rules.

Consequently, a physics-driven strategy based on electromagnetic travel-time differences, rather than amplitude magnitude, is essential for reliable and quantitative detection. By explicitly exploiting the time delay (Δt) between reflections from the sleeper top and the ballast surface, the proposed method establishes a direct geometric relationship between wave propagation time and void height. Leveraging the centimeter-level resolution of high-frequency GPR (≥1.5 GHz), this study develops a time-domain peak-searching framework as the primary detection mechanism, with CWT employed solely for auxiliary verification, thereby reducing sensitivity to environmental variability and migrated waveform uncertainty.

The primary contributions are threefold:

1. A problem-oriented GPR interpretation framework is developed for sleeper void detection, in which conventional signal-processing tools are reorganized according to the physical mechanism of support loss, rather than used as generic visualization or anomaly-extraction modules. An integrated detection framework is developed with time-domain peak searching as the core method, complemented with CWT for verifying time delays (Δt) and void heights (*H*), thereby enabling the direct quantitative interpretation of shallow support-loss defects from electromagnetic travel-time differences.
2. Automated and quantitative void assessment. Through the integration of adaptive thresholding and auxiliary sleeper localization, the method achieves a fully automated identification of void locations, spatial extents, and severity levels. This capability supports real-time, vehicle-mounted inspections without disrupting railway operations.
3. Comprehensive validation framework. The proposed method is rigorously validated via gprMax numerical simulations and field experiments, providing robust empirical evidence of superior performance compared with vibration-based techniques and conventional GPR methods.

Collectively, these contributions fill a critical gap in existing NDT practices, offering a practical, high-precision solution for proactive railway track maintenance.

## 2. Materials and Methods

### 2.1. Principle of GPR-Based Sleeper Void Detection

The detection principle of the proposed method is based on the electromagnetic reflection characteristics of high-frequency GPR in ballasted tracks. Under normal conditions, the ballast layer provides uniform support to the sleeper underside, with the ballast surface typically 5–10 cm above the sleeper base. Sleeper voids, also known as hanging sleepers, commonly arise from a localized ballast loss or degradation, resulting in a reduced ballast surface elevation beneath or adjacent to the sleeper. Consequently, an increased apparent vertical height difference is evident between the sleeper top, which serves as a stable reference, and the ballast surface interface.

**Figure 1:**
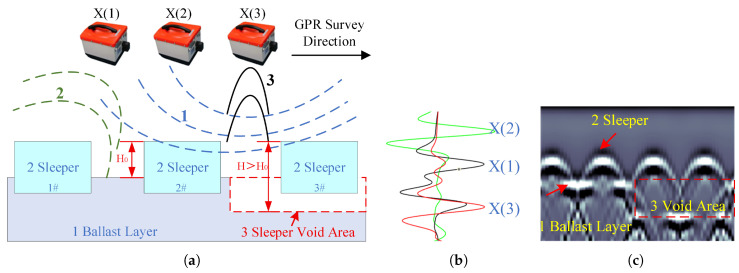
Schematic of GPR wave response on sleeper void area in ballast track. (**a**) GPR survey on ballast bed. (**b**) A-Scans. (**c**) B-Scan.

As shown in Figure 1, high-frequency GPR transmits electromagnetic waves that propagate through the air and penetrate the track structure. Strong reflections occur at dielectric discontinuities, particularly at interfaces such as the air–ballast boundary and the sleeper top and bottom surfaces.

The primary reflection from the sleeper top remains relatively stable, while the reflection from the ballast surface is shifted to a later arrival time in the time domain due to the reduced surface elevation (i.e., increased wave travel time to and from the interface). By comparing the time delay (Δt) between the peak reflection from the sleeper top, $t_{s}$, and that from the ballast surface, *t*, in A-scan waveforms, the relative height difference, *H*, can be calculated as:

(1) $$H=\frac{(t-t_{s})\times v}{2}$$

where *v* denotes the electromagnetic wave velocity (adjusted for dielectric permittivity, approximately 0.3 m/ns in air or calibrated, according to ballast conditions).

An adaptive threshold, $H_{0}$ (typically sleeper thickness, e.g., 18 cm for concrete sleepers, plus compensation for normal ballast height and moisture), is then applied: if $H\geq H_{0}$, the sleeper is identified as voided. This strategy exploits the inherent physical relationship between sleeper voids and adjacent ballast deficiency, enabling a quantitative assessment without direct imaging of the void gap beneath the sleeper.

The theoretical relationship between the vertical resolution of a GPR antenna and its operating frequency is intrinsically related to the electromagnetic wave’s wavelength (λ).

(2) $$\lambda =\frac{c}{f\times \sqrt{\varepsilon_{r}}}$$

where $\varepsilon_{r}$ is the relative dielectric constant of the medium, *c* is the wave velocity in air, c=0.3m/ns, and *f* is the antenna center frequency in Hz.

The vertical resolution of GPR systems is commonly approximated as δ≈λ/4−λ/10, indicating that higher antenna frequencies yield a finer resolution. For sleeper and ballast track materials, typical values of $\varepsilon_{r}$ are 9 for concrete sleepers and 5 for dry ballast, respectively; these can increase (e.g., to 10–20) in wet or fouled conditions, shortening the effective wavelength and reducing resolution.

In China, the nominal central thickness of concrete sleepers is commonly 18 cm, and hanging gaps associated with sleeper voids are typically on the order of a few centimeters. Consequently, to accurately resolve such shallow features, high-frequency antennas are preferred [20], achieving centimeter-level vertical resolution in ballast media.

### 2.2. Experiment Design

The experimental design of this study consists of two complementary components: a numerical experiment and a field experiment. These experiments are conducted to validate the proposed high-frequency GPR-based method for detecting sleeper voids in ballasted railway tracks by evaluating its accuracy, robustness, and practical applicability. The numerical experiment focuses on simulating GPR signal responses under controlled conditions, thereby enabling parametric optimization and quantitative error analysis using synthetic models such as gprMax, in contrast, the field experiment assesses the method’s performance in real-world environments by accounting for environmental factors, including ballast fouling and moisture, and by verifying its suitability for vehicle-mounted inspection applications.

#### 2.2.1. Numerical Experiment

The objective of this numerical experiment is to establish a baseline for void detection accuracy and to optimize signal processing parameters under idealized conditions.

**Figure 2:**
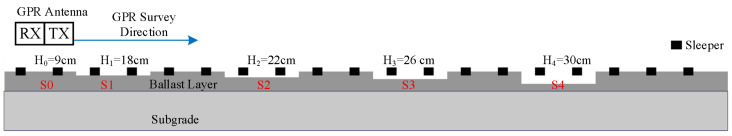
Numerical model of sleeper void in ballasted railway track.

As illustrated in Figure 2, a finite-difference time-domain (FDTD) model is constructed to represent a typical ballasted railway track section with concrete sleepers (18 cm in thickness with a 60 cm spacing between adjacent sleepers), a ballast layer (30 cm thick), and a subgrade layer (100 cm thick). Four sleeper void scenarios are incorporated into the model from area S1–S4, where the void gap heights between the sleeper underside and the ballast surface are 0, 4, 8 and 12 cm, respectively. A reference condition representing a normally supported ballasted track is defined between the sleeper top and the ballast layer is $H_{0}=9$ cm, corresponding to half of the sleeper thickness, and serving as the threshold for void identification. A GPR antenna with a central frequency of 1.5 GHz is positioned 30 cm above the sleeper surface. The detailed simulation parameters are summarized in Table 1. The dielectric constants used in the numerical model were selected based on previously reported measurements for railway track materials. The void region beneath the sleeper was modeled as an air-filled gap with a relative dielectric constant, $\varepsilon_{r}\approx 1$, and $\varepsilon_{r}$ of the ballast layer and the subgrade were set to 5 and 8, respectively, according to the previous laboratory and field measurements [3,31]. Concrete sleepers typically exhibit relative permittivity values ranging from approximately 6 to 9, and a representative value of $\varepsilon_{r}=9$ was adopted for the sleeper in this study.

#### 2.2.2. Field Data Acquisition

**Figure 3:**
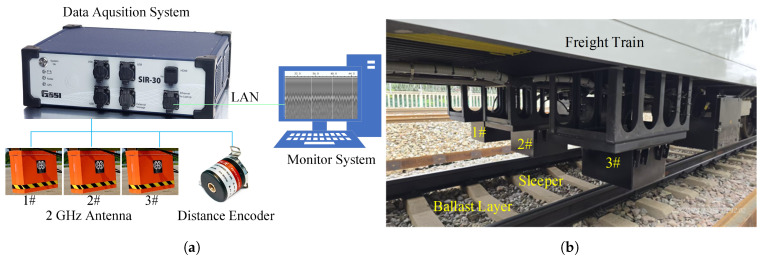
GPR field survey on ballast railway track. (**a**) GPR data acquisition system. (**b**) GPR antenna mounted on the freight train.

To validate the numerical simulation results, field surveys were conducted along a freight railway line in China. A four-channel GPR data acquisition system (GSSI SIR30, Geophysical Survey Systems, Inc., Nashua, NH, USA) was employed, and three 2 GHz air-coupled horn antennas were mounted on a freight train to scan the track centreline and rail shoulders, as illustrated in Figure 3, respectively. The antenna was positioned approximately 30 cm above the sleeper surface.

The train-mounted GPR system was integrated with a high-resolution distance encoder for georeferencing. The system supports a maximum operating speed of 120 km/h; the trace interval was set to 5 cm to ensure adequate spatial sampling density. The sampling frequency was 34.13 GHz with a 15 ns time window, resulting in 512 sampling points per trace. Data were collected at a travel speed of 80 km/h and stored in .dzt file, after which the multi-channel data were separated into individual channels for further data analysis. In this study, only the GPR data from the central antenna was used.

## 3. Automated Assessment of Sleeper Void

### 3.1. GPR Signal Process

**Figure 4:**
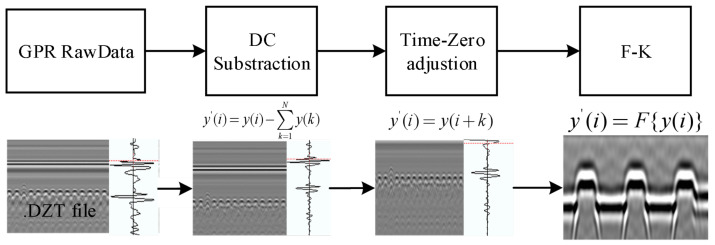
GPR signal process protocol for sleeper void.

High-frequency GPR signals produce strong reflections at shallow dielectric interfaces, such as the sleeper top and the ballast surface, while undergoing pronounced attenuation and scattering within the ballast layer. These signal characteristics are critical for sleeper void detection, as localized ballast loss induces distinct time shifts in the reflected signals from surface interfaces. Nevertheless, raw GPR data are frequently contaminated by instrumental drift, direct-wave interference, and hyperbolic clutter arising from scattered reflections. To reliably extract the time delay (Δt) between the peak reflections from the sleeper top and the ballast surface peaks for void height estimation, a dedicated signal processing pipeline was developed, as illustrated in Figure 4.

The signal processing pipeline comprises three core steps, which are sequentially applied to the raw dataset A[ns,ntr].

#### 3.1.1. DC Offset Subtraction

Instrumental bias introduces baseline shifts across traces. The mean value of each A-scan trace is subtracted to center the signal:

(3) $$B\left[:,n_{\mathrm{tr}}\right]=A\left[:,n_{\mathrm{tr}}\right]-\frac{1}{n_{s}}\sum_{i=1}^{n_{s}}A\left[i,n_{\mathrm{tr}}\right]$$

where *i* denotes the sample index, ns is the total sample points for A-scan trace, and ntr represents the trace number.

#### 3.1.2. Time-Zero Adjustment

**Figure 5:**
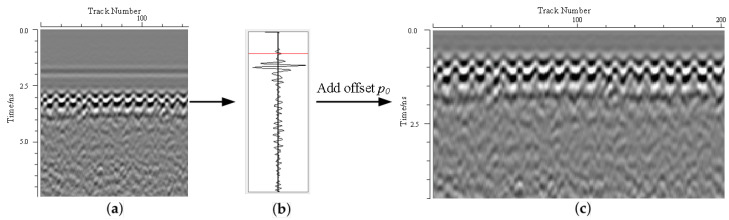
Time-zero adjustment flowchart. (**a**) Before zero adjustment. (**b**) Search peak value. (**c**) After zero adjustment.

Air-coupled acquisition produces a strong direct wave typically exhibiting a Ricker wavelet shape that obscures early shallow reflections. A synthetic Ricker wavelet template is generated and cross-correlated with each trace to locate the direct-wave peak (*p*). A fixed offset, $p_{0}$ = 10 samples (optimized for f≥1.5 GHz antennas), is applied to suppress sidelobes and align the subsequent signal window, as illustrated in Figure 5:

(4) $$C\left[n,n_{\mathrm{tr}}\right]=B\left[p+p_{0},n_{\mathrm{tr}}\right]$$

where *p* is the sample point corresponding to the direct-wave, $p_{0}$ is the offset point, and *n* is the remaining number of samples in the A-scan trace.

The Ricker template is defined as:

(5) $$r\left(t\right)=\left(1-2\pi^{2}f_{0}^{2}t^{2}\right)e^{-\pi^{2}f_{0}^{2}t^{2}}$$

where $f_{0}$ is the central frequency of the GPR antenna.

#### 3.1.3. Frequency-Wavenumber (F-K) Migration

Diffraction hyperbolas from sleepers and scatterers degrade lateral resolution and reduce interface continuity. F-K migration is employed to collapse these hyperbolas and enhance layer focusing. The algorithm applies a phase shift in the frequency-wavenumber domain using a constant velocity, v=0.3 m/ns over the shallow time window t=[0,6] ns; detailed descriptions of the F-K migration algorithm can be found in Refs. [20,32,33].

(6) $$D\left[n,n_{\mathrm{tr}}\right]=F^{-1}\left\{C\left[n,n_{\mathrm{tr}}\right]\right\}$$

Collectively, these processing steps yield sharpened B-scan images suitable for accurate peak picking.

### 3.2. CWT

GPR reflections from dielectric interfaces, such as the sleeper top or ballast surface, appear in the time domain as localized, high-amplitude impulses resembling Ricker wavelets in the time domain. The CWT is employed as a supplementary analysis tool for peak detection in noisy or cluttered A-scans, complementing the primary direct time-domain peak-searching. CWT provides superior time-frequency localization through scale-dependent windowing (constant-Q analysis), unlike fixed-window methods such as the short-time Fourier transform. This enables the effective characterization of transient reflection impulses across multiple resolutions.

The CWT of a GPR signal s(t) is defined as:

(7) $$WT_{x}\left(b,a\right)=<x\left(t\right),\psi_{a,b}\left(t\right)\rangle =\frac{1}{\sqrt{a}}\int_{-\infty }^{+\infty }x\left(t\right)\psi^{*}\left(\frac{t-b}{a}\right)dt$$

where x(t) denotes the GPR signal, and $\psi_{a,b}\left(t\right)$ is the mother wavelet—typically a complex Morlet wavelet for optimal matching to Ricker-like reflections). *a* is the scale parameter, *b* is the translation, and $\psi^{*}$ denotes the complex conjugate.

These reflection impulses produce concentrated energy maxima in the scalogram due to abrupt signal changes. Identifying the global maximum in the magnitude scalogram |WT(a,b)| reliably locates the interface arrival time, even under heavy clutter or waveform distortion, because signal energy is coherently aggregated across multiple scales, while incoherent noise is effectively suppressed.

### 3.3. Sleeper Void Detection Algorithm

Unlike generic commercial GPR software, which mainly provides standard functions such as preprocessing, radargram visualization, migration, and manual or semi-automatic signal interpretation, the present study implements a task-specific workflow for sleeper void detection. The workflow is centered on the physical time-delay difference between the sleeper-top reflection and the ballast-surface reflection, followed by height conversion, adaptive threshold-based classification, and consecutive-trace refinement. Therefore, the proposed method is not merely a direct use of standard software functions but also a problem-oriented interpretation framework designed for the quantitative identification of sleeper support loss.

**Figure 6:**
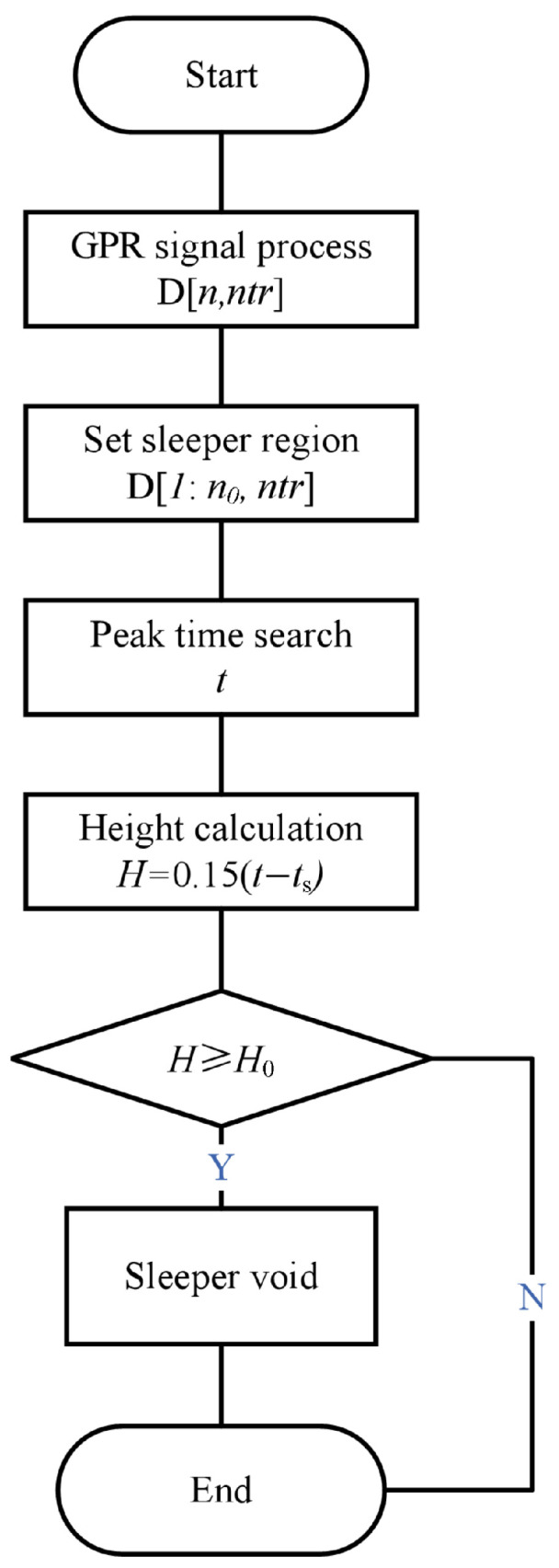
Sleeper void detection flow chart.

The sleeper void detection algorithm was refined based on experimental findings, prioritizing time-domain peak searching with polarity consideration due to its superior accuracy and robustness while employing the CWT as a supplementary verification tool in complex scenarios. The overall workflow of the sleeper void detection algorithm is illustrated in Figure 6.

Step 1: The raw GPR data matrix A[ns,ntr] is processed according to Figure 4, and the processed GPR data was stored in matrix D[n,ntr].

Step 2: The sleeper top region is identified, and the corresponding arrival time at sleeper top is extracted as $t_{s}$.

Step 3: Peak time search. The primarily peak detection is performed using a time-domain amplitude-based method with a positive polarity selection (as simulations and field survey employ negative Ricker-wavelet excitation, yielding positive surface responses) to find max positive amplitudes in A-scans. For verification in noisy signals, CWT-based analysis is applied by transforming the signal to time-frequency domain and getting the amplitude of the CWT result as $WT=\left|\mathrm{CWT}(D[:,J])\right|$, as well as finding the max energy at Time_idx to select as the ballast surface reflection time, t, for each trace.

Step 4: Height estimation: $H=0.15(t-t_{s})$, with ts as the sleeper top time. (Note: Accounts for the effective electromagnetic wave velocity 0.3/2 m/ns and the one-way travel path).

Step 5: Thresholding: Adaptive $H_{0}$ = sleeper thickness (e.g., 18 cm). If $H\geq H_{0}$, classified as voided; merged to determine the spatial position and longitudinal extent.

## 4. Results and Discussion

### 4.1. Numerical Analysis

A numerical model was developed to simulate a ballasted railway track with an 18 cm sleeper thickness, 60 cm sleeper spacing, a normal ballast–sleeper separation of 9 cm, and four void regions. The numerical experiment was carried out using gprMax3.0 with a spatial grid resolution of 0.02 m, and the scanning interval was set to 5 cm. The transmitting and receiving antennas were separated at 10 cm apart and positioned 0.3 m above the sleeper surface. A 20 ns time window was employed to capture reflections from sleepers to the ballasted layer.

**Figure 7:**
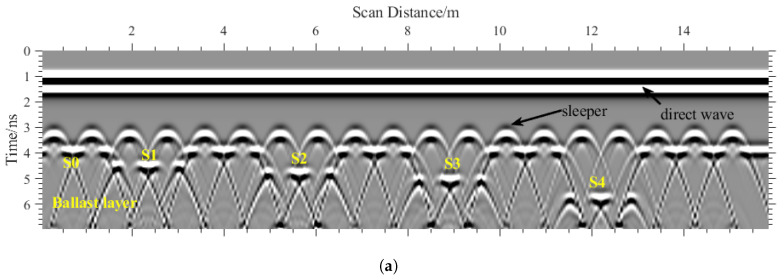

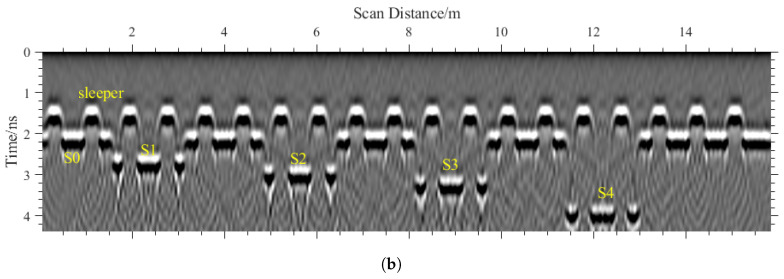
Numerical simulation result. (**a**) Original result. (**b**) Procced result.

The simulated results are presented in Figure 7, where 19 sleepers are clearly identified by prominent hyperbolic signatures; the ballast surface and the four predefined void regions were also observed. However, the voids directly beneath the sleepers are not distinctly visible, which motivates the proposed strategy of indirectly detecting sleeper voids by locating the ballast surface for assistance in detecting the sleeper void. To visualize the void area more clearly, a time-zero adjustment and F-K migration were applied to the simulated data, and Figure 7b resulted in clearer target delineation. The hyperbolic features were suppressed, and the ballast layer top was flatter than that in Figure 7a; an increasing void thickness leads to a deeper apparent ballast interface in B-scan.

### 4.2. Comparison of Peak Search Methods: CWT Versus Time-Domain Amplitude

To optimize peak detection for identifying sleeper tops, ballast surfaces, and void interfaces, the CWT against a direct time-domain amplitude-based method was first evaluated.

**Figure 8:**
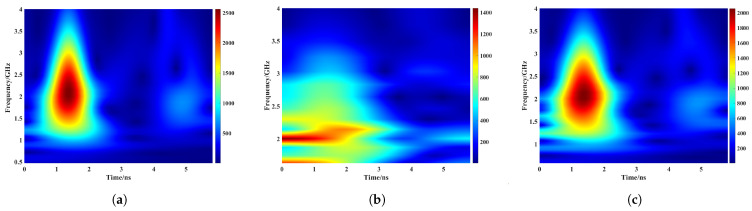
Compared CWT results with three different wavelet functions. (**a**) Morlet. (**b**) Bump. (**c**) Morse.

Prior to formal comparison, a preliminary assessment of mother wavelet functions was conducted using representative A-scan signals from sleeper regions after F-K migration. Three commonly employed wavelets were tested: (a) analytic Morlet, (b) Bump, and (c) Generalized Morse. As illustrated in Figure 8, the Bump wavelet exhibited poor energy concentration, yielding dispersed scalogram coefficients that hindered reliable localization of the ballast surface reflection. In contrast, the analytic Morlet and Generalized Morse wavelets achieved more focused energy maxima. The analytic Morlet wavelet, however, provided the sharpest peaks and superior alignment with interface arrivals, attributed to its optimal matching with GPR reflection impulses. Consequently, it was selected for subsequent CWT analyses.

**Figure 9:**
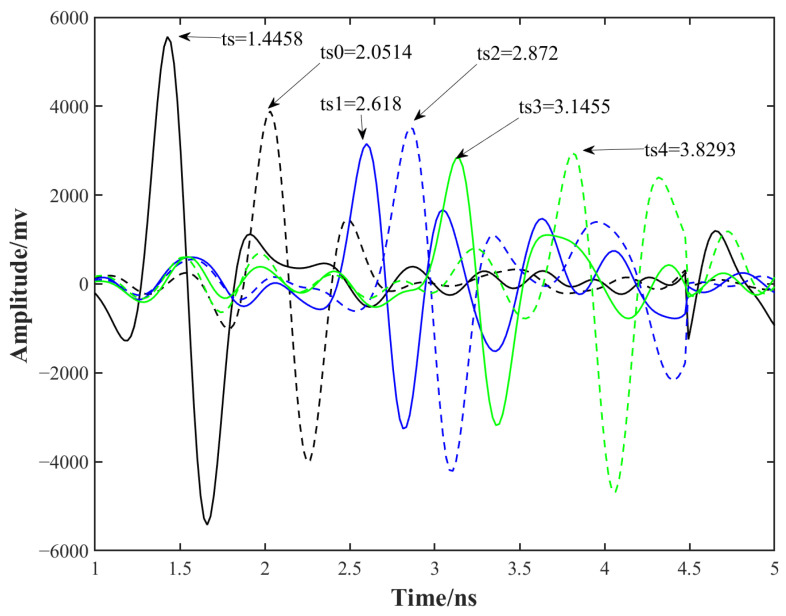
Peak time search for the sleeper top and ballast surface by positive Ricker match.

For peak localization, a positive-polarity criterion was consistently applied in both methods; this choice is justified by the use of a negative Ricker wavelet for excitation, which produces positive-polarity surface reflections. The time-domain amplitude method identifies interface arrivals by directly searching for the maximum positive amplitudes in the A-scans. The results are summarized in Figure 9, where the detected peak arrival times correspond to the sleeper top, the normal ballast layer (S0), and four simulated void areas (S1–S4), occurring at 1.4458 ns, 2.0514 ns, 2.6180 ns, 2.8720 ns, 3.1455 ns, and 3.8293 ns, respectively.

The corresponding time delays relative to the sleeper are 0.6057 ns, 1.1722 ns, 1.4262 ns, 1.6997 ns, and 2.3835 ns. Under the assumption of an electromagnetic wave velocity of 0.3 m/ns in air, the corresponding height differences are calculated to be 9.08 cm, 17.58 cm, 21.39 cm, 25.50 cm, and 35.75 cm. Compared with the ground-truth numerical model shown in Figure 2, the detection errors are 0.08 cm, −0.42 cm, 0.61 cm, 0.50 cm, and 5.75 cm, respectively. The largest error occurs in the severe void case (S4, 12 cm hanging height), which is likely attributable to increased signal attenuation and enhanced multiple-scattering effects associated with deeper air gaps.

**Figure 10:**
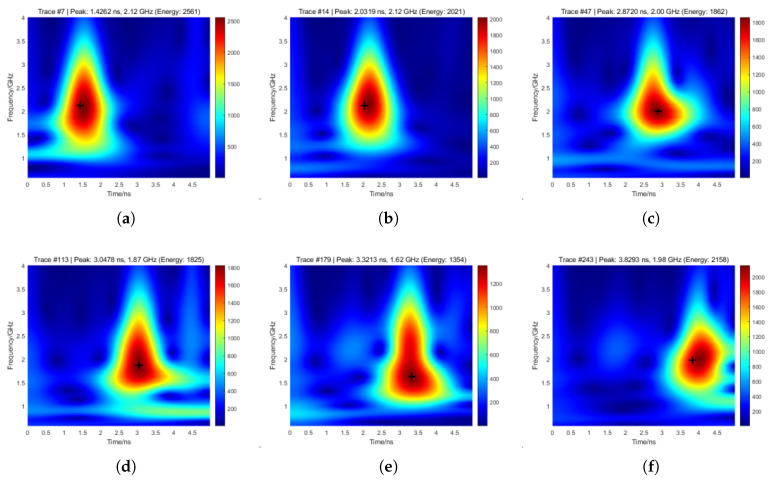
Peak detection results using the CWT method. (**a**) Sleeper. (**b**) Normal ballasted layer (S0). (**c**) S1. (**d**) S2. (**e**) S3. (**f**) S4.

In parallel, the CWT-based method employing the analytic Morlet wavelet was applied to the same post-F-K migration signals, with positive-polarity masking used to isolate physically meaningful energy maxima and suppress spurious responses (Figure 10).

The detected peak arrival times were 1.5434 ns for the sleeper top, 2.1491 ns for the normal ballast layer (S0), and 2.8720 ns, 3.0478 ns, 3.3213 ns, and 3.8293 ns for the four simulated void regions (S1–S4), respectively. The corresponding height estimates were 9.09 cm, 19.93 cm, 22.57 cm, 26.67 cm, and 34.29 cm, yielding detection errors of 0.09 cm, 1.93 cm, 0.57 cm, 0.67 cm, and 2.29 cm relative to the numerical model. All simulated void regions were correctly identified using an adaptive threshold of $H_{0}=18$ cm.

To clarify the practical selection of the primary peak-picking strategy, the error statistics were further analyzed by grouping void scenarios into shallow–moderate cases (S0–S3) and an extreme severe case (S4). As shown in Table 2, the time-domain peak-searching method achieves substantially lower errors for shallow–moderate voids (MAE = 0.40 cm; RMSE = 0.45 cm), whereas the CWT-based method exhibits larger deviations in these typical scenarios (MAE = 0.82 cm; RMSE = 1.04 cm).

In contrast, for the severe void case (S4), the CWT method yields a smaller error (2.29 cm vs. 5.75 cm). This behavior may be associated with its multi-scale representation capability, which can partially capture dispersed reflection energy under stronger attenuation and multiple scattering conditions.

Although the overall averaged error of the CWT method (1.11 cm) is slightly lower than that of the time-domain method (1.30 cm), this average is strongly influenced by the severe void case. From an engineering perspective, most sleeper voids encountered in ballasted tracks are shallow and correspond to early-stage support loss, where accurate discrimination near the decision threshold is most critical for preventive maintenance. In these typical shallow-void conditions, the time-domain method demonstrates superior precision and stability.

Therefore, the direct time-domain amplitude-based peak search is adopted as the primary detection strategy. It offers consistent alignment with physical interface arrivals, lower sensitivity to migration-induced waveform distortions, reduced sensitivity to phase-related waveform variations, and lower computational complexity without requiring wavelet selection or scale optimization. The CWT method is retained as an auxiliary verification tool in complex or extreme cases. Both approaches are capable of identifying void presence; however, the time-domain strategy provides better reliability for routine shallow-void detection in practical railway inspection scenarios.

Future refinements may consider hybrid implementations in which time-domain detection is complemented by selective CWT verification under severe or high-noise conditions.

These results indicate that the contribution of the present study is not the introduction of a new standalone signal-processing operator, but the establishment of a practical detection framework that selects and organizes conventional tools according to the physical characteristics of sleeper voids and the requirements of quantitative field inspection.

### 4.3. Field Experiment

A field survey was conducted on the Shuohuang Railway in Hebei Province, China, to validate the proposed method under realistic operating conditions. This heavy-haul freight corridor has been in service for over two decades and primarily transports coal using C80 wagons with axle loads of 25 t, assembled into trains with total weights of approximately 20,000 t. A 2000 m test section containing known sleeper voids, previously confirmed through manual inspection, was selected for evaluation. The track section includes a mixture of intact and voided sleepers, with ballast fouling conditions ranging from clean to moderately fouled, thereby providing a representative testing environment for practical railway operations.

**Figure 11:**
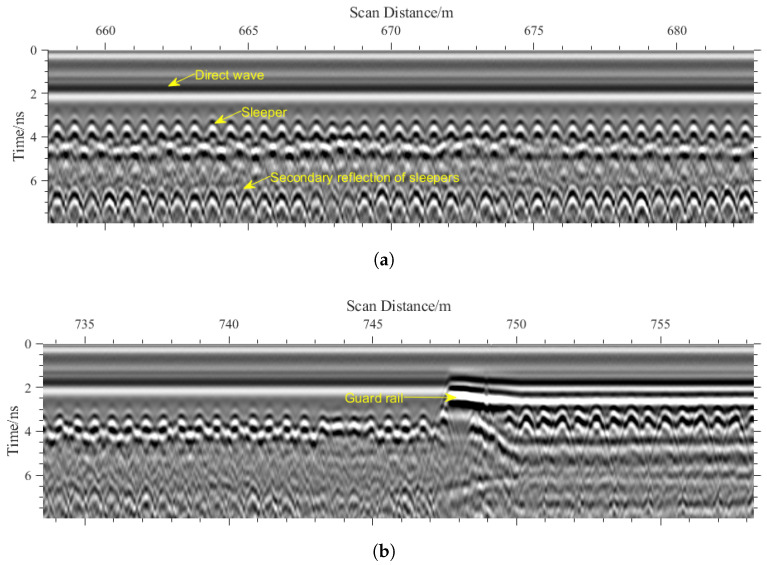
Typical B-scan features of ballasted track. (**a**) Ballasted track with secondary reflection. (**b**) Guard rail.

Typical B-scan characteristics obtained from the surveyed section are shown in Figure 11. The direct wave is clearly observable above the sleeper regions, accompanied by pronounced hyperbolic diffraction patterns. As illustrated in Figure 11a, secondary reflections are visible beneath the sleepers; however, these reflections do not correspond to the true sleeper bottom interfaces. In contrast, Figure 11b shows no such secondary reflections but clearly delineates the guard rail region.

**Figure 12:**
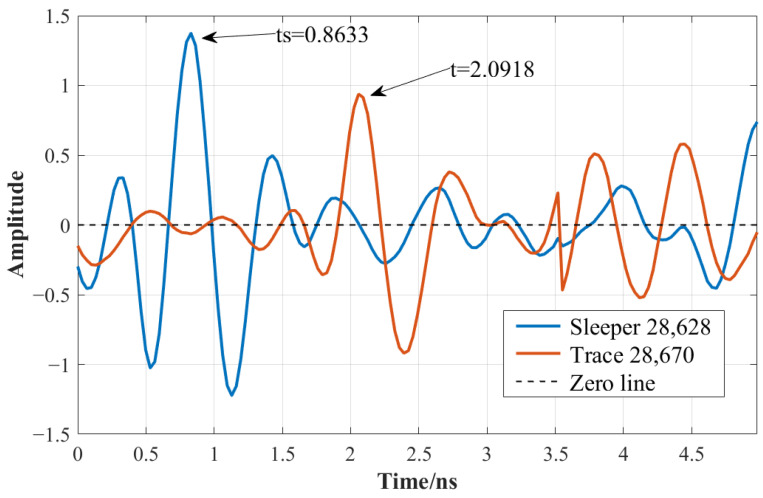
Peak time search between sleeper and void area in time domain.

**Figure 13:**
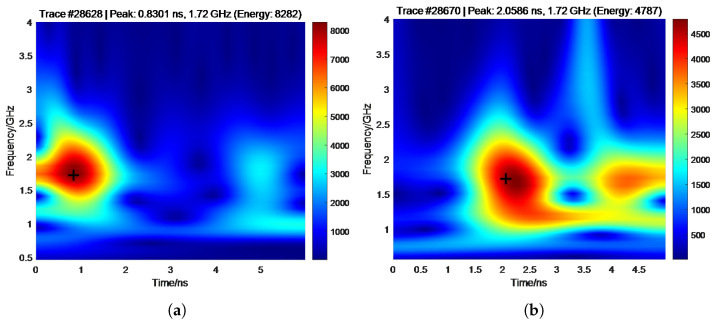
CWT results for the sleeper region and the void area. (**a**) Sleeper. (**b**) Void area.

To evaluate the proposed void detection method, processed GPR data from a track segment located at approximately 1420 m were analyzed. Two representative A-scan traces were selected for comparison: one corresponding to a sleeper region (trace 28,628) and one from a ballast region (trace 28,670). Peak arrival times were extracted using both the time-domain amplitude-based method and the CWT-based approach, resulting in an identical height difference, *H*, of 18.43 cm for both approaches (Figure 12 and Figure 13). As this value exceeds the adaptive threshold (e.g., 18 cm), the analyzed location was classified as a sleeper void, demonstrating strong consistency between the two detection techniques.

**Figure 14:**
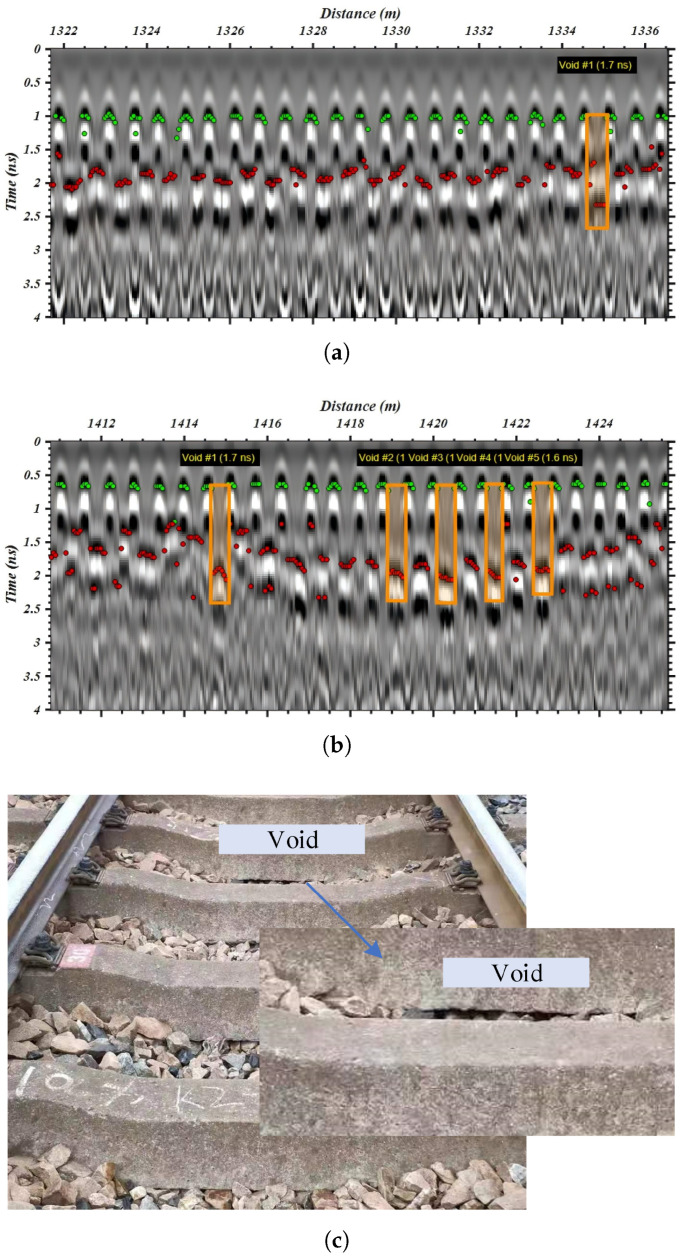
Validation of sleeper void detection results. (**a**) Case 1: void area. (**b**) Case 2: void area. (**c**) Field validation of Case 2 at 1419 m.

For broader validation, two test sections with 300-trace segment were analyzed using the time-domain peak-search method. To clearly visualize the detection results, the detection results were divided with two groups, where the upper layer was marked with a green point for the sleeper, and the lower layer was marked with a red point for the ballast layer. Height differences satisfying H≥18 cm were retained; thus, the time difference Δt≥1.2 ns would be determined as a void area. The detection results were overlapped on the corresponding B-scan (Figure 14a,b), enabling a clear delineation of sleeper void regions. A comparison with on-site visual inspections confirmed that this section exhibits significant ballast deficiency (Figure 14c), with visible hanging sleepers, thereby substantiating the effectiveness of the proposed method.

Notably, several isolated detections can be observed in Figure 14a at around 1416 m and 1424 m. Given the 60 cm spacing between adjacent sleepers, which corresponds to approximately 12 traces at a spatial sampling interval of 5 cm, a refinement criterion was introduced, whereby a void is confirmed only if at least 4 consecutive traces within this interval satisfy the detection threshold. This post-processing rule effectively suppresses false positives and enhances the robustness of the proposed method for practical applications.

### 4.4. Comparison with Existing GPR-Based Void Detection Approaches

GPR has been widely used for detecting subsurface defects in railway and pavement structures. Over the past decades, various GPR-based approaches have been developed to identify voids, delamination, and other structural anomalies. These methods can generally be classified into three categories: reflection-feature-based methods, B-scan image analysis methods, and machine-learning-based approaches.

The first category includes methods based on reflection amplitude or waveform characteristics, where voids are identified by analyzing abnormal reflections caused by impedance contrasts between materials [34]. Such methods are relatively straightforward and computationally efficient, and they have been widely applied in pavement void detection and subsurface anomaly identification. However, their performance is often sensitive to signal attenuation, material heterogeneity, and environmental noise. As a result, the detection results may become unstable in complex field conditions where signal reflections are strongly affected by ballast structure irregularities.

The second category involves B-scan image-based analysis, where defects are identified through geometric or texture features in radar images. This strategy has been successfully applied in many GPR applications, such as detecting buried objects or pavement defects. However, the applicability of B-scan-based methods to hanging-sleeper detection is limited. In railway ballast structures, sleepers generate strong radar reflections that dominate the B-scan images. During the visualization process, radar signals are often compressed or interpolated into image representations, which can obscure subtle structural variations beneath the sleeper. Because hanging-sleeper features are relatively small and easily interfered with by sleeper reflections, they often fail to form stable or continuous patterns in B-scan images. Consequently, purely image-based approaches may not reliably detect hanging-sleeper regions.

More recently, machine learning and deep learning methods have been introduced for automatic defect detection in GPR data [35,36]. These approaches can achieve high detection accuracy when sufficient labeled data are available and have shown promising results in pavement damage detection and underground object recognition. However, these data-driven approaches are primarily designed for pattern recognition or target classification problems, where defects exhibit distinguishable radar signatures. In contrast, hanging-sleeper detection is essentially a structural interface identification problem. In engineering practice, a hanging sleeper is defined based on the height difference between the sleeper bottom and the ballast surface, typically using a threshold criterion (e.g., 18 cm). Therefore, the key task is to accurately determine the relative positions of these structural interfaces, rather than to classify radar image patterns. Without explicitly incorporating this physical relationship, purely data-driven models may identify signal features but cannot directly reflect the geometric constraint that defines the defect.

In this study, the proposed method explicitly incorporates the physical geometric relationship between the sleeper and ballast layers. By extracting the elevation positions of the two interfaces and evaluating their height difference, together with spatial continuity constraints across adjacent ballast points, hanging-sleeper regions can be identified directly, according to engineering definitions. This physics-informed framework improves detection robustness by reducing false detections caused by local signal fluctuations or noise. In addition, the method provides clear physical interpretability, which is important for practical railway inspection applications.

To better position the proposed method within the context of existing GPR-based void detection techniques, the characteristics of representative approaches are summarized in Table 3.

## 5. Conclusions

This study has developed a novel high-frequency GPR-based method for detecting sleeper voids in ballasted railway tracks, achieving high accuracy and robustness through a combination of time-domain analysis and CWT-based techniques. The proposed approach demonstrates clear advantages for real-time railway maintenance by reducing safety risks and operational costs. The main conclusions are summarized as follows:

1. The proposed method primarily employs time-domain peak searching with polarity consideration, supplemented by CWT-based analysis for verification. By exploiting the time delay between reflections from the sleeper top and the ballast surface, the approach enables the quantitative estimation of sleeper void heights.
2. Compared with traditional vibration-based approaches and conventional GPR techniques, the proposed method exhibits superior robustness against common interferences such as ballast fouling and moisture. It allows an automated and quantitative assessment of void locations, extents, and severities.
3. The method is well suited for real-time, vehicle-mounted inspections, addressing a critical requirement for proactive maintenance in high-speed and heavy-haul railway systems by minimizing operational disruptions and safety risks.

Despite these advancements, several limitations remain. The method relies on high-frequency antennas (≥1.5 GHz), which may experience reduced penetration depth under severely fouled or wet ballast conditions, potentially degrading detection performance in extreme environments. In addition, the field validation was conducted on a single railway line, which may limit the generalizability of the results to other regions with different ballast compositions and loading conditions.

Future research will focus on hybrid time–frequency analysis strategies, such as combining time-domain methods with advanced CWT variants, to further enhance noise resilience. Additional efforts will include integrating convolutional neural network (CNN) models for automated sleeper localization and multi-ensor data fusion, as well as expanding field trials under extreme conditions, including high moisture content and frozen ballast. These developments are expected to further improve detection precision and broaden the applicability of the proposed method for sustainable railway infrastructure management.

## Figures and Tables

**Table 1:** Parameters of the FDTD model.

Component	H × W Dimension (m)	Dielectric Constant
Air	1.0 × 16.0	1
void	(0.0,0.04,0.08,0.12)×1.2	1
ballast layer	0.3 × 16.0	5
sleeper	0.22 × 0.18	9
subgrade	1.0 × 16.0	8

**Table 2:** Error statistics for time-domain and CWT methods (unit: cm).

Severity Group	Cases	Metric	Time-Domain	CWT
Shallow–Moderate	S0–S3	MAE	0.40	0.82
		RMSE	0.45	1.04
		Max abs. error	0.61	1.93
Severe	S4	Abs. error	5.75	2.29

**Table 3:** Comparison of representative GPR-based detection methods.

Methods	Detection Principle	Advantages	Limitations
Reflection-feature-based methods	Identify abnormal reflections or amplitude changes in radar signals	Simple implementation and low computational cost	Sensitive to noise, material heterogeneity, and signal attenuation
B-scan image analysis methods	Detect geometric or texture features in B-scan radar images	Intuitive visualization of subsurface structures	Strong sleeper reflections may obscure small void features; unstable feature continuity
Machine learning/deep learning methods	Learn defect patterns from labeled radar data	High detection accuracy with sufficient training data	Requires large annotated datasets and lacks explicit physical interpretability
Proposed method	Detect voids based on elevation difference between sleeper and ballast layers with spatial continuity constraints	Physically interpretable and consistent with engineering definition of hanging sleepers	Requires reliable extraction of structural interfaces

## Data Availability

The data used to support the findings of this study are included within the article.

## Abbreviations

The following abbreviations are used in this manuscript:

GPR	Ground-Penetrating Radar
CWT	Continuous Wavelet Transform
NDT	Non-Destructive Testing
FDTD	Finite-Difference Time-Domain
CNN	Convolutional Neural Network
